# Real-world data on adult AML with *FLT3*-ITD mutation from the Thai acute leukemia working group

**DOI:** 10.3389/fonc.2025.1606943

**Published:** 2025-08-29

**Authors:** Thanawat Rattanathammethee, Ekarat Rattarittamrong, Chinadol Wanitpongpun, Smith Kungwankiattichai, Weerapat Owattanapanich, Chantiya Chanswangphuwana, Chantana Polprasert, Thanakrit Piyajaroenkij, Pimjai Niparuck, Supawee Saengboon, Wasithep Limvorapitak, Panachai Silpsamrit, Kannadit Prayongratana, Chantrapa Sriswasdi, Jakrawadee Julamanee, Pirun Saelue, Aimwipa Sasakul, Dusit Jit-ueakul, Chajchawan Nakhakes, Adisak Tantiworawit

**Affiliations:** ^1^ Division of Hematology, Department of Internal Medicine, Faculty of Medicine, Chiang Mai University, Chiang Mai, Thailand; ^2^ Division of Hematology, Department of Internal Medicine, Faculty of Medicine, Khon Kaen University, Khon Kaen, Thailand; ^3^ Division of Hematology, Department of Medicine, Faculty of Medicine, Siriraj Hospital, Mahidol University, Bangkok, Thailand; ^4^ Division of Hematology and Center of Excellence in Translational Hematology, Faculty of Medicine, Chulalongkorn University, Bangkok, Thailand; ^5^ Division of Hematology, Department of Medicine, Ramathibodi Hospital, Mahidol University, Bangkok, Thailand; ^6^ Division of Hematology, Department of Internal Medicine, Thammasat University, Pathumthani, Thailand; ^7^ Division of Hematology, Department of Internal Medicine, Phramongkutklao Hospital and College of Medicine, Bangkok, Thailand; ^8^ Hematology Unit, Division of Internal Medicine, Prince of Songkla University, Hat Yai, Songkhla, Thailand; ^9^ Division of Hematology, Department of Medicine, Chulabhorn Hospital, Bangkok, Thailand; ^10^ Division of Hematology, Department of Medicine, Faculty of Medicine, Vajira Hospital, Navamindradhiraj University, Bangkok, Thailand; ^11^ Division of Hematology, Department of Medicine, Rajavithi Hospital, Bangkok, Thailand

**Keywords:** FLT3-ITD mutation, acute myeloid leukemia, real-world data, Thailand, treatment outcomes

## Abstract

**Background:**

*FLT3*-ITD mutations are among the most common genetic alterations in acute myeloid leukemia (AML) and are associated with poor clinical outcomes. However, data from low- and middle-income countries remain limited. This study aimed to investigate the prevalence, clinical characteristics, treatment patterns, and outcomes of adult AML patients with *FLT3*-ITD mutations in Thailand.

**Methods:**

We analyzed data from 360 adult patients with newly diagnosed AML, prospectively collected from 11 institutions nationwide between 2016 and 2023. *FLT3*-ITD mutational status, clinical features, response to therapy, and survival outcomes were compared between *FLT3*-ITD and *FLT3*-wild-type patients.

**Results:**

*FLT3*-ITD mutations were detected in 28.1% of patients. *FLT3*-ITD patients had higher white blood cell counts, bone marrow blast percentages, and NPM1 co-mutations compared to wild-type *FLT3*. Induction chemotherapy rates were similar, but FLT3 inhibitor use was nearly absent. Complete remission was achieved in 55.7% of *FLT3*-ITD patients versus 66.5% in wild-type *FLT3*. Median overall survival was significantly shorter in the *FLT3*-ITD group (8.8 vs. 13.2 months, p=0.039), while relapse-free survival was not significantly different. Multivariable analysis confirmed *FLT3*-ITD mutation as an independent predictor of poor overall survival.

**Conclusions:**

In this nationwide real-world study, *FLT3*-ITD AML was associated with inferior outcomes despite comparable induction therapy. Limited access to FLT3-targeted treatments and stem cell transplantation may contribute to these disparities. Our findings highlight the urgent need for expanding access to molecular testing and targeted therapies in resource-limited settings.

## Introduction

Acute myeloid leukemia (AML) is a heterogeneous hematological malignancy characterized by rapid proliferation of myeloid progenitor cells in the bone marrow, leading to impaired hematopoiesis and subsequent clinical manifestations. The prognosis for AML patients is influenced by various factors, including age, cytogenetic abnormalities, and specific genetic mutations. Among these, the *FLT3*-ITD (Fms-like tyrosine kinase 3 internal tandem duplication) mutation has emerged as a critical prognostic factor. This mutation occurs in approximately 18-30% of AML cases and is associated with poor prognosis, characterized by higher rates of relapse and reduced overall survival compared to patients without this mutation (1–4).

FLT3 is a receptor tyrosine kinase that plays a significant role in hematopoiesis and cell survival. The presence of *FLT3*-ITD mutation leads to constitutive activation of the FLT3 signaling pathway, promoting cell proliferation and survival while inhibiting apoptosis. Consequently, *FLT3*-ITD mutations are linked to a more aggressive disease course, with patients often presenting with higher white blood cell counts and increased percentages of bone marrow blasts. Studies have consistently shown that *FLT3*-ITD mutations correlate with inferior outcomes in patients with AML, including lower complete remission rates and shorter overall survival (4, 5).

The significance of *FLT3*-ITD indicates prognosis and alters treatment approaches, making it a mutational test for classifying AML subtypes according to the World Health Organization’s definition of the disease in 2022 (WHO classification of hematolymphoid tumors, 5th edition: Myeloid and Histiocytic/Dendritic neoplasms) (6). A study in Thailand by the Thai Acute Leukemia Working Group (TALWG), which compiles patient data from multicenter medical institutions across Thailand, reported that information on adult AML patients, focusing only on risk group classification based on chromosomal abnormalities, showed 1-year and 2-year overall survival rates of 31.9% and 29.6%, respectively (7). There is yet to be a report on gene mutations used for classification and prognostic indications. Additionally, there has been no analysis of AML patient data regarding the prevalence of *FLT3* mutations, clinical characteristics, other co-occurring chromosomal abnormalities, treatment regimens, or detailed survival rates.

*FLT3* mutations can be detected at diagnosis and upon disease relapse. Two types of *FLT3* mutations have been identified: internal tandem duplication (*FLT3*-ITD), found in 20-30% of cases, and mutations in the kinase domain (*FLT3*-TKD), found in 7-12% (2, 8–10). Targeted therapies for patients with *FLT3* mutations, such as *FLT3* tyrosine kinase inhibitor (TKI) drugs, have clear benefits in terms of increasing response rates and overall survival. Studies involving AML patients with *FLT3* mutations at diagnosis, using standard high-dose chemotherapy in combination with *FLT3* TKIs such as midostaurin (RATIFY study) (11), sorafenib (SORAML study) (12), and quizartinib (QuANTUM-First study) (13), have shown significantly improved outcomes in terms of relapse-free survival and overall survival compared to standard chemotherapy alone. In cases of relapsed or refractory (R/R) AML with *FLT3* mutations, the use of TKI compared to high-dose chemotherapy alone, such as gilteritinib (ADMIRAL study) (14) and quizartinib (QuANTUM-R study) (15), has also shown significant improvements in overall survival. This demonstrates the critical role of targeted therapy in treatment. However, access to these therapies remains limited in many regions, including Thailand, where healthcare disparities may affect treatment availability and patient outcomes.

The present study aimed to assess the prevalence and clinical outcomes of *FLT3*-ITD mutations in adult AML patients in Thailand using data from the Thai Acute Leukemia Working Group (TALWG) registry. By analyzing real-world data, this study sought to provide insights into the clinical characteristics, treatment patterns, and survival outcomes of *FLT3*-ITD AML patients in a setting with limited access to targeted therapies. Furthermore, this study contributes to the growing body of evidence regarding the impact of *FLT3*-ITD mutations on clinical outcomes in AML, particularly in the context of real-world practice in Thailand. By elucidating the prevalence and implications of this mutation, our findings may serve as a foundation for future research and policy development aimed at improving the management of AML in the region.

## Methods

### Study participants and definitions

This study conducted a multicenter prospective observational analysis using data from the Thai Acute Leukemia Working Group (TALWG) registry, covering the period from January 1, 2016, to December 31, 2023. The study included adult patients aged ≥ 18 years with newly diagnosed AML and available *FLT3*-ITD status. The clinical characteristics, cytogenetics, mutational status, risk stratification based on the European Leukemia Net (ELN) 2022 AML risk classification (4), treatment regimens (according to guideline for diagnosis and management of adult acute leukemia on behalf of the Thai Society of Hematology) (16), response rates, percentage of allogeneic stem cell transplantation, and survival outcomes, including relapse-free survival (RFS) and overall survival (OS), were collected and analyzed. RFS was defined as the time from AML diagnosis to relapse and OS was defined as the time from diagnosis to death from any cause. Patients with acute promyelocytic leukemia (APL), mixed phenotypic acute leukemia (MPAL), or lacking clinical information including *FLT3* mutational status and survival outcomes were excluded from the study. *FLT3*-ITD detection techniques vary across institutions, including polymerase chain reaction (PCR) based on capillary electrophoresis and next-generation sequencing (NGS) of myeloid panels. NGS was used to determine other genetic mutation testing for AML risk classification. High-intensity treatment consisted of standard induction chemotherapy using the 7 + 3 regimen, which included a seven-day continuous intravenous infusion of cytarabine at 100 mg/m² over 24 h, combined with a three-day bolus of idarubicin at 12 mg/m². Alternatively, a modified 5 + 2 regimen was used (17), consisting of a five-day continuous infusion of cytarabine at 100 mg/m² over 24 hours and a two-day bolus of idarubicin at 12 mg/m². This was followed by 3–4 cycles of consolidation chemotherapy, involving intermediate-dose cytarabine (IDAC) at 1.5 g/m² or high-dose cytarabine (HiDAC) at 3 g/m², administered intravenously every 12 hours for three days. Low-intensity treatment included hypomethylating agents, such as a seven-day course of subcutaneous azacitidine at 75 mg/m², hydroxyurea, or transfusion support, as needed. Palliative care in this study was the only transfusion support as needed. Some AML patients with relapsed or refractory disease may receive salvage treatment with MEC (a five-day regimen consisting of intravenous Mitoxantrone 8 mg/m², Etoposide 100 mg/m², and Cytarabine 1 g/m²) or FLAG-Idarubicin (Fludarabine 30 mg/m² and Cytarabine 2 g/m² intravenously for five days, along with Idarubicin 8 mg/m² intravenously for three days, and G-CSF).

### Objectives

The primary endpoint of this study was the prevalence of the *FLT3*-ITD mutation in adult patients with AML in Thailand. The secondary endpoints included the clinical characteristics, treatment patterns, response rates, and survival outcomes of AML patients with and without the *FLT3*-ITD mutation in a real-world setting in Thailand.

### Statistical analysis

Continuous variables are summarized using median values and range or interquartile range (IQR), while categorical variables are presented as percentages. The Chi-square test or Fisher’s exact test was used to compare categorical variables between *FLT3*-ITD AML and wild-type *FLT3* AML patients, whereas the Mann-Whitney U test was used for continuous variables. Survival analysis was performed using the Kaplan-Meier method with the log-rank test to determine differences between groups. The Cox proportional hazard model was used to evaluate the hazard ratio (HR) with 95% confidence interval (95% CI) in the time-to-event analysis of the survival outcomes for univariable analyses (p-value <0.1 were included in the multivariable analyses. The sample size calculation was based on the primary outcome of the expected prevalence of the *FLT3*-ITD mutation in adult AML patients, which was estimated at 30% (1). A sample size of 360 patients was required to achieve a margin of error of 5% in estimating the prevalence with a 95% CI, accounting for a potential 10% loss. With this sample size, the anticipated 95% CI was 25-35% (18).

## Results

### Clinical characteristics

A total of 360 adult patients with newly diagnosed AML were included in this study. *FLT3*-ITD detection methods consisted of PCR in 140 cases (38.9%) and NGS in 220 cases (61.1%). Among these, 101 patients (28.1%) had *FLT3*-ITD mutations, whereas 259 patients (71.9%) had wild-type *FLT3*. The median age of patients with *FLT3*-ITD AML was 52 years (IQR 41-59) of compared to 51 years (IQR 36-61) for those with wild-type *FLT3* AML (p=0.247). The proportion of male patients was similar between the *FLT3*-ITD (41.5%) and wild-type *FLT3* (48.3%) groups (p=0.254). Regarding the Eastern Cooperative Oncology (ECOG) performance status, a higher proportion of patients with *FLT3*-ITD AML had an ECOG score of 3–4 compared than those with wild-type *FLT3* AML (6.9% vs. 1.9%, p=0.018). Other baseline characteristics, such as the AML subtype, showed no significant differences between the two groups. Patients with *FLT3*-ITD AML had significantly higher median white blood cell counts (44.5 x 10^9^/L, IQR 12.0-110.1) compared to those with wild-type *FLT3* AML (23.5 x 10^9^/L, IQR 7.1-62.1) (p=0.002). The median bone marrow blast percentage was also higher in the *FLT3*-ITD group (85%, IQR 68.7-90.0) compared than in the wild-type *FLT3* group (80%, IQR 50.0-90.0) (p=0.011). Normal cytogenetics was more prevalent in the *FLT3*-ITD group (69.3%) than in the wild-type *FLT3* group (52.1%) (p=0.003). There were significant differences in the distribution of certain mutations between the two groups. The *FLT3*-ITD group had a higher prevalence of *NPM1* mutations (45.6% vs. 12.3%, p<0.001) and a lower incidence of biallelic *CEBPA* mutations (0 vs. 8.5%, p=0.043) (**Table 1**). There were two types of *NPM1* mutations: type A (c.863_864insTCTG) was found in 71.7% and 65.6% of *FLT3*-ITD and wild-type *FLT3* while type B (c.863_864insCATG) was found in 28.3% of *FLT3*-ITD and 34.4% of wild-type *FLT3* AML.

**Table 1:** Clinical characteristics between *FLT3*-ITD AML and wild-type *FLT3* AML.

Characteristics	Total (n = 360)		p-value
	*FLT3*-ITD AML (n = 101)	Wild-type *FLT3* AML (n = 259)	
Age, years	52 (41-59)	51 (36-61)	0.247
Age range, n (%)			
<30 years	12 (11.9)	42 (16.2)	0.301
30–60 years	65 (64.3)	151 (58.3)	0.292
>60 years	24 (23.8)	66 (25.5)	0.735
Male sex, n (%)	42 (41.5)	125 (48.3)	0.254
ECOG performance status, n (%)			
0-1	73 (72.3)	206 (79.5)	0.138
2	21 (20.8)	48 (18.5)	0.625
3-4	7 (6.9)	5 (1.9)	0.018
Subtype of AML, n (%)			
* de novo* AML	86 (85.1)	208 (80.3)	0.286
Secondary AML	15 (14.9)	51 (19.7)	0.363
AML-MR	9 (8.9)	32 (12.4)	0.355
t-AML	1 (0.9)	2 (0.7)	0.838
Previous diagnosed MDS or MPN	5 (4.9)	17 (6.6)	0.566
White blood cell count, x 10^9^/L	44.5 (12.0-110.1)	23.5 (7.1-62.1)	0.002
Hemoglobin, g/dL	7.4 (6.0-9.4)	7.9 (6.7-9.4)	0.074
Platelets, x 10^9^/L	55 (34-94)	50 (24-91)	0.265
Bone marrow blasts, %	85 (68.7-90.0)	80 (50-90)	0.011
Cytogenetics, n (%)			
Normal	70 (69.3)	135 (52.1)	0.003
Other intermediate*	26 (25.7)	54 (20.9)	0.316
11q23-rearranged	1(0.9)	7 (2.7)	0.322
-5/5q-	0	2 (0.7)	0.376
-7/7q-	1 (0.9)	13 (5.0)	0.076
Monosomal karyotype	0	13 (5.0)	0.022
Complex	2 (1.9)	30 (11.6)	0.004
t(8;21)	1 (0.9)	17 (6.6)	0.029
inv(16)	0	4 (1.5)	0.209
Mutations, n (%)			
* NPM1*	46 (45.6)	32 (12.3)	<0.001
* biallelic CEBPA*	0/32	5/59 (8.5)	0.043
* TP53*	0/23	7/51 (13.7)	0.127
European LeukemiaNet 2022 risk, n (%)			
Favorable	9 (8.9)	53 (20.5)	0.009
Intermediate	87 (86.1)	151 (58.3)	<0.001
Adverse	5 (4.9)	55 (21.2)	<0.001

Continuous variables are reported as median (interquartile range). AML-MR, Acute myeloid leukemia with myelodysplasia; t-AML, therapy-related acute myeloid leukemia; MDS, Myelodysplastic neoplasm; MPN, Myeloproliferative neoplasm.*Other intermediate: *FLT3*-ITD AML (n = 26): +8 (6 cases)/+13 (6 cases)/t(7;11)(p15;p15) (2 cases)/and one case each of: +1q, t(3;13), +8 + 22, +8 inv(9), -9q, +11, t(12;17), -13, -16, +21, +22, -22 and Wild-type *FLT3* AML (n = 54): +8 (14 cases)/+21 (5 cases)/+4 (4 cases)/-Y (4 cases)/t(7;11) (3 cases)/and one case each of: -1q, t(1;12)(q21;q24.3) with t(16;21), -2, +3, t(3;5), +5, +5 + 8, +5 inv(9), -5-12, -6, -7, inv(8), +8-9q, -9q, -12-20, t(13;14), t(x;15)(p22.3;q15), +11, t(11;18), inv(21), +13, inv(17), -16-20, -X.

### Treatment allocation and response rates

Treatment intensity was guided by physician discretion, largely based on age and performance status. Patients receiving high-intensity therapy were significantly younger (median age: 44 years; IQR 32–54) than those receiving low-intensity therapy (median age: 65 years; IQR 59–70) (p < 0.001). The proportion of patients with ECOG performance status 0–2 was higher in the high-intensity group (98.9%) compared to the low-intensity group (90.9%) (p = 0.001). Among *FLT3*-ITD patients, 69.3% received high-intensity induction chemotherapy, resulting in a complete remission (CR) rate of 55.7%. In the wild-type *FLT3* group, 73.8% received high-intensity therapy, with a CR rate of 66.5%. These differences were not statistically significant (p = 0.397 and p = 0.109, respectively). Primary refractory disease occurred in 27.1% of *FLT3*-ITD patients and 16.7% of wild-type patients (p = 0.061). Among those receiving low-intensity regimens, non-response was common: 74.2% in *FLT3*-ITD and 60.3% in wild-type *FLT3* (p = 0.180). In this study consisted of 45 patients who underwent allogeneic stem cell transplantation in the real-world data setting in Thailand. Of these, 26 patients (57.8%) received upfront allogeneic stem cell transplantation after first complete remission (CR1) while 19 patients (42.2%) underwent transplantation after achieving second complete remission (CR2) salvage therapy for relapse or refractory disease. Among those transplanted in CR1, there were 5 patients with *FLT3*-ITD AML and 21 with wild-type *FLT3* AML. For those transplanted in CR2, 9 patients had *FLT3*-ITD AML and 10 had wild-type *FLT3* AML.

### Overall survival

**Figure 1:**
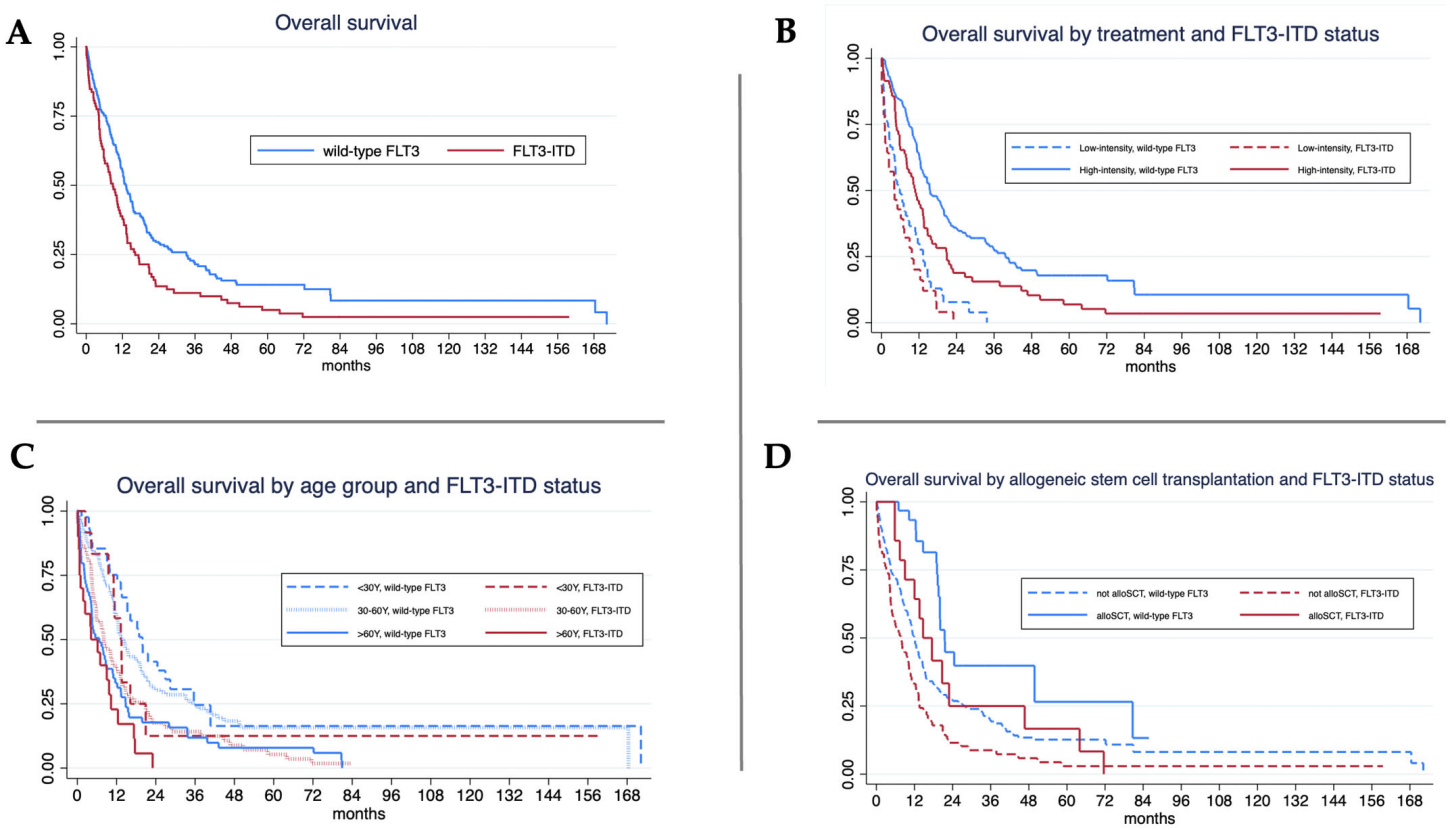
Overall survival (OS) analysis. **(A)** *FLT3*-ITD mutant showed significant lower in median OS 8.8 vs. 13.2 months (log-rank test, p < 0.001).; **(B)** For high-intensity treatment, *FLT3*-ITD mutant showed significant lower in median OS 10.8 vs. 15.5 months (log-rank test, p = 0.002) while the median OS of *FLT3*-ITD and wild-type were 4.1 vs. 5.9 months (log-rank test, p = 0.169) in patients with low-intensity treatment.; **(C)** For age < 30 years, median OS of *FLT3*-ITD and wild-type were 13.3 vs. 19.9 months (log-rank test, p = 0.137) while *FLT3*-ITD mutant showed significant lower in median OS 8.1 vs. 13.9 months (log-rank test, p = 0.001) in patients aged 30–60 years and 4.2 vs. 6.7 months (log-rank test, p = 0.148) in patients aged older than 60 years.; **(D)** For allogeneic stem cell transplantation (alloSCT), median OS of *FLT3*-ITD and wild-type were 14.7 vs. 21.5 months (log-rank test, p = 0.019). *FLT3*-ITD mutant also showed significant lower in median OS 7.8 vs. 12 months (log-rank test, p<0.001) in those without alloSCT (log-rank test, p = 0.148).

Median follow-up was shorter in *FLT3*-ITD patients (8.1 months, IQR 3.4–14.8) than in wild-type *FLT3* patients (11.4 months, IQR 4.6–21.5) (p = 0.006). Overall survival (OS) was significantly lower in *FLT3*-ITD patients (median 8.8 months) compared to wild-type *FLT3* (13.2 months, log-rank test p < 0.001) (**Table 2**, **Figure 1A**). When stratified by treatment intensity, the median OS in the high-intensity treatment group was 10.8 months for *FLT3*-ITD and 15.5 months for wild-type *FLT3* patients (p = 0.002). In the low-intensity group, the median OS was 4.1 months for *FLT3*-ITD and 5.9 months for wild-type *FLT3* patients (p = 0.169). In both mutational groups, high-intensity therapy resulted in significantly longer OS than low-intensity treatment (p < 0.001 for each comparison) (**Figure 1B**). When analyzed by age, a significant OS difference was observed among patients aged 30–60 years, with median OS of 13.9 months in wild-type *FLT3* compared to 8.1 months in *FLT3*-ITD (p = 0.001) (**Figure 1C**). Among patients who underwent allogeneic stem cell transplantation, OS was longer in the wild-type *FLT3* group than in the *FLT3*-ITD group (21.5 vs. 14.7 months, p < 0.001). Transplantation was associated with significantly improved OS in both subgroups: in *FLT3*-ITD patients, median OS was 14.7 months with transplantation versus 7.8 months without (p < 0.001); in wild-type *FLT3* patients, the corresponding OS was 21.5 vs. 12.0 months (p < 0.001) (**Figure 1D**).

**Table 2:** Treatment patterns and survival outcomes between patients with *FLT3*-ITD AML and wild-type *FLT3* AML.

Characteristics	Total (n = 360)		p-value
	*FLT3*-ITD AML (n = 101)	Wild-type *FLT3* AML (n = 259)	
High-intensity treatment	70 (69.3)	191 (73.8)	0.397
7 + 3	64/70 (91.4)	165/191 (86.4)	0.388
5 + 2	6/70 (8.6)	26/191 (13.6)	0.422
CR rate	39/70 (55.7)	127/191 (66.5)	0.109
Refractory	19/70 (27.1)	32/191 (16.7)	0.061
Low-intensity treatment	31 (30.7)	68 (26.3)	0.431
HMAs	12/31 (38.7)	25/68 (36.7)	0.686
Palliative care	19/31 (61.3)	43/68 (63.3)	0.657
CR rate	3/31 (9.7)	10/68 (14.7)	0.492
Refractory	23/31 (74.2)	41/68 (60.3)	0.180
Ara-C Consolidation	44 (62.9)	130 (68.1)	0.429
IDAC	25/44 (56.8)	86/130 (66.2)	0.281
HiDAC	19/44 (43.2)	44/130 (33.9)	0.265
Allogeneic stem cell transplantation	14 (13.9)	31 (11.9)	0.626
Relapse	36 (35.6)	81 (31.3)	0.132
Follow up time, months	8.1 (3.4-14.8)	11.4 (4.6-21.5)	0.006
Overall survival (OS), months	8.8 (4.2-16.2)	13.2 (6.5-33.2)	<0.001
Treatment			
High-intensity treatment	10.8 (4.7-20.9)	15.5 (8.8-39.7)	0.002
Low-intensity treatment	4.1 (1-9.7)	5.9 (2.1-13.4)	0.169
Age group			
<30 years	13.3 (9.5-16.2)	19.9 (12.2-35.6)	0.137
30–60 years	8.1 (4.3-20.7)	13.9 (7.9-36.0)	0.001
>60 years	4.2 (0.7-10.3)	6.7 (2.2-14.8)	0.148
Allogeneic stem cell transplantation			
Yes	14.7 (7-22.9)	21.5 (19.1-80.7)	0.019
No	7.8 (3.1-13.6)	12 (4.6-28)	<0.001
Relapse-free survival (RFS), months	7.3 (5.2-11.7)	7.8 (4.9-11.3)	0.704
Treatment			
High-intensity treatment	7.4 (5.3-11.7)	7.6 (4.9-11.4)	0.764
Low-intensity treatment	5.2 (4.7-6.2)	7.8 (3.2-9.1)	0.717
Age group			
<30 years	7.5 (6.5-11.7)	8 (5.8-14.9)	0.823
30–60 years	7.4 (5.3-11.8)	7.2 (4.6-10.8)	0.829
>60 years	5.2 (4.7-6.2)	7.8 (7.2-11)	0.033
Allogeneic stem cell transplantation			
Yes	5.6 (4.7-6.4)	10.3 (7.6-12)	0.013
No	7.5 (5.3-11.7)	7.4 (4.8-11)	0.456

Continuous variables were reported as median (interquartile range). CR, complete remission; HMAs, Hypomethylating agents.

### Relapse-free survival

**Figure 2:**
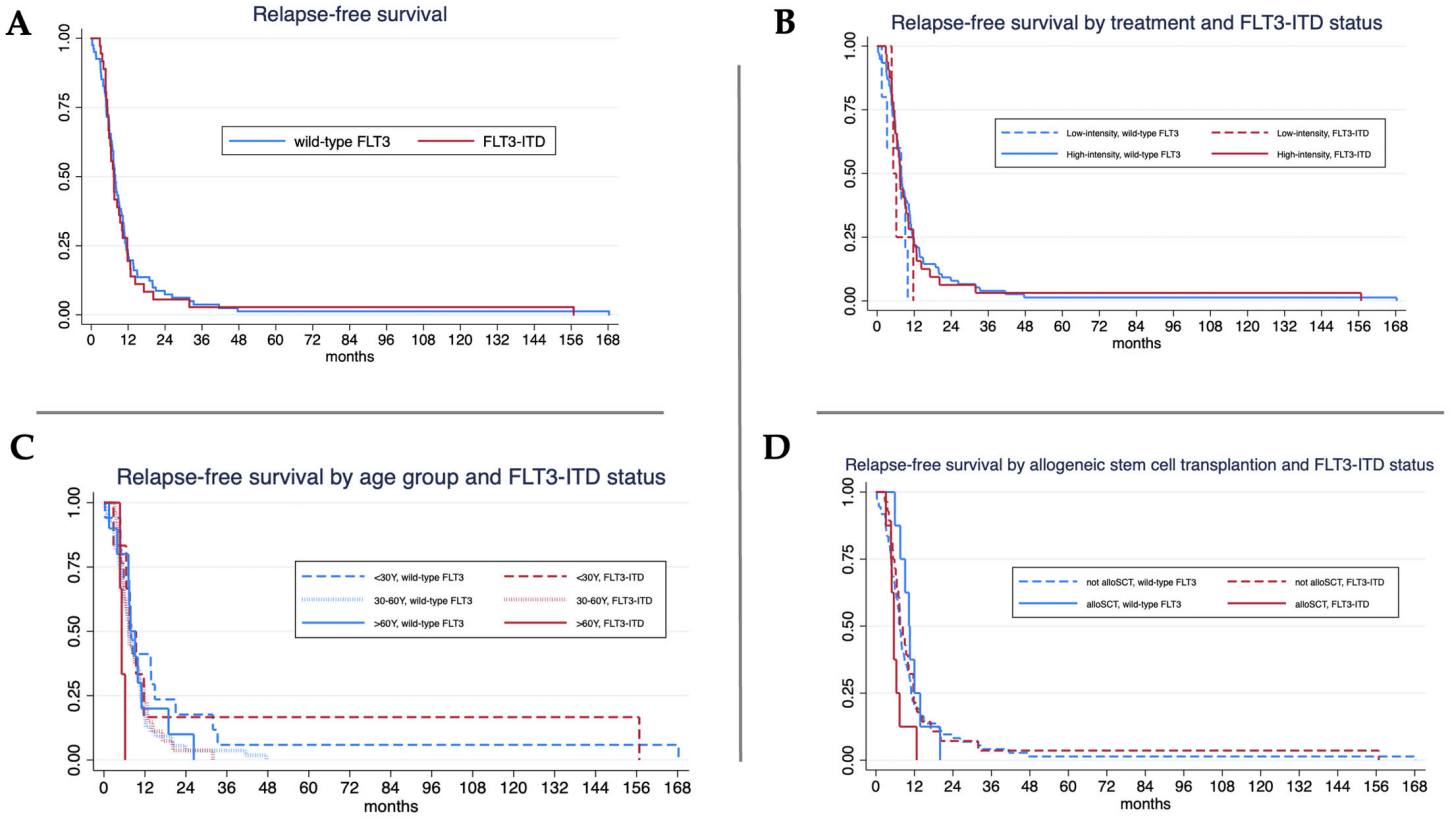
Relapse-free survival (RFS) analysis. **(A)** Median RFS in *FLT3*-ITD vs. wild-type *FLT3* was 7.3 vs. 7.8 months (log-rank test, p = 0.704).; **(B)** For high-intensity treatment, *FLT3*-ITD mutant and wild-type FLT3 AML showed median OS 7.4 vs. 7.6 months (log-rank test, p = 0.764) and the median RFS of *FLT3*-ITD and wild-type were 5.2 vs. 7.8 months (log-rank test, p = 0.717) in patients with low-intensity treatment.; **(C)** For age < 30 years, median RFS of *FLT3*-ITD and wild-type were 7.5 vs. 8 months (log-rank test, p = 0.823) and 7.4 vs. 7.2 months (log-rank test, p = 0.829) in patients aged 30–60 years*. FLT3*-ITD mutant showed significant lower in median RFS 5.2 vs. 7.8 months (log-rank test, p = 0.033) in patients aged older than 60 years.; **(D)** For allogeneic stem cell transplantation (alloSCT), median RFS of wild-type *FLT3* was longer than *FLT3*-ITD of 10.3 vs. 5.6 months (log-rank test, p = 0.013).

Relapse occurred in 117 patients (32.5%): 35.6% in FLT3-ITD and 31.3% in wild-type *FLT3* (p = 0.132). Median relapse-free survival (RFS) was comparable between the two groups: 7.3 months for *FLT3*-ITD and 7.8 months for wild-type *FLT3* (log-rank test, p = 0.704) (**Figure 2A**). When stratified by treatment intensity, RFS was 7.4 months in *FLT3*-ITD and 7.6 months in wild-type *FLT3* among patients receiving high-intensity therapy (p = 0.764), and 5.2 months versus 7.8 months, respectively, among those receiving low-intensity treatment (p = 0.717). No significant differences in RFS were observed within each intensity group (**Figure 2B**). In the subgroup of patients aged over 60 years, *FLT3*-ITD AML was associated with significantly shorter RFS compared to wild-type *FLT3* (5.2 vs. 7.8 months, p = 0.033) (**Figure 2C**). Among patients who underwent allogeneic stem cell transplantation, wild-type *FLT3* patients had a significantly longer median RFS than those with *FLT3*-ITD (10.3 vs. 5.6 months, p = 0.013). However, when assessed within each mutational subgroup, the impact of transplantation on RFS did not reach statistical significance (**Figure 2D**).

### Subgroup and multivariable analyses

**Figure 3:**
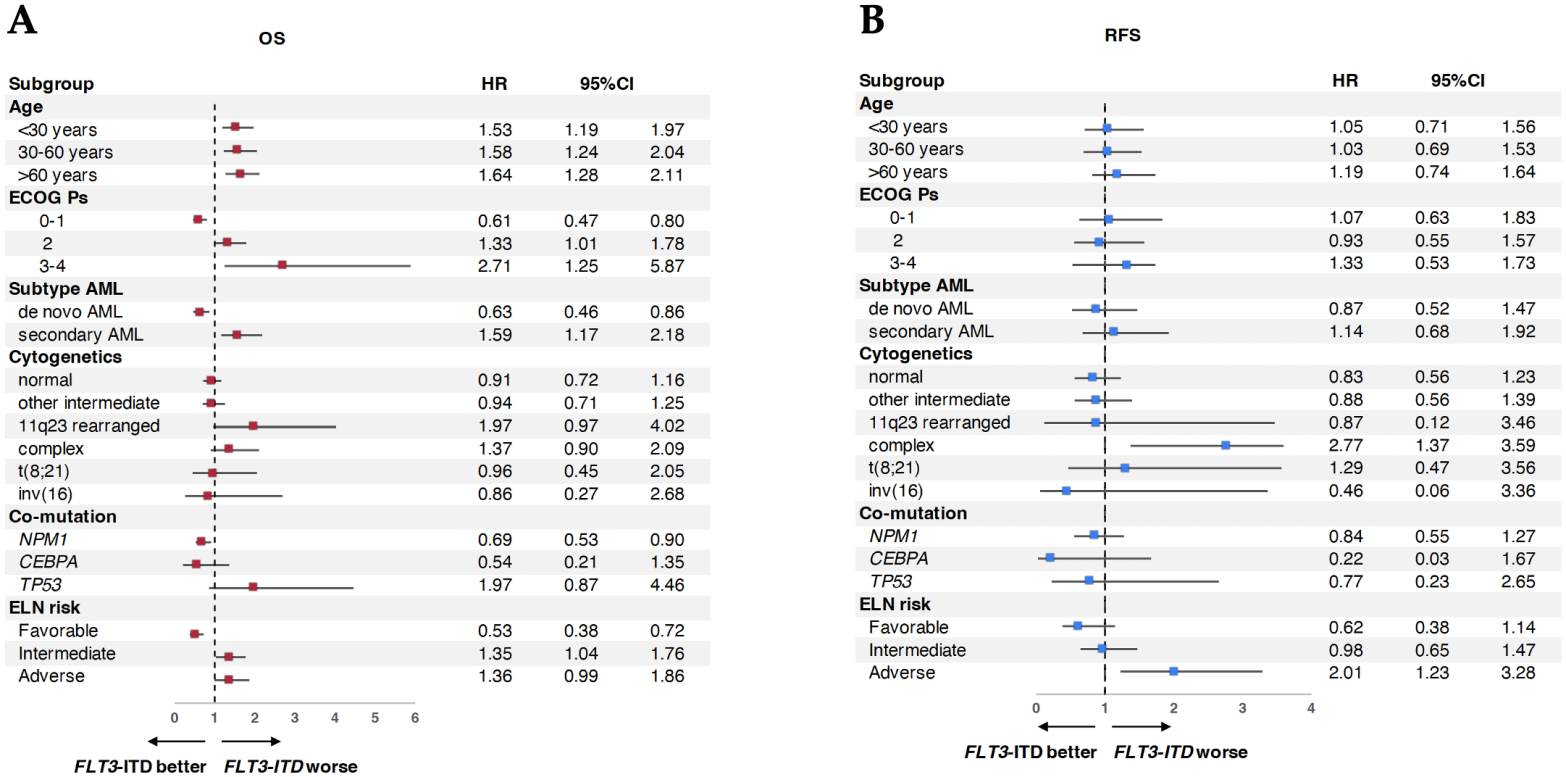
Subgroup analysis of AML patients between *FLT3*-ITD vs. wild-type *FLT3* on survival. **(A)** Subgroup analysis of overall survival (OS). **(B)** Subgroup analysis of relapse-free survival (RFS).

Subgroup analyses showed that *FLT3*-ITD conferred a significantly higher risk of death across all age groups (**Figure 3A**). ECOG 3–4 was associated with a worse prognosis (HR 2.71, 95% CI: 1.25–5.87), as was secondary AML (HR 1.59, 95% CI: 1.17–2.18) and 11q23 rearrangement (HR 1.97, 95% CI: 0.97–4.02). Favorable outcomes were observed in patients with co-mutated *NPM1* (HR 0.69, 95% CI: 0.53–0.90), while *TP53* mutations trended toward poorer OS (HR 1.97, 95% CI: 0.87–4.46). For RFS, complex cytogenetics (HR 2.77, 95% CI: 1.37–3.59) and adverse ELN risk (HR 2.01, 95% CI: 1.23–3.28) were associated with increased relapse risk (**Figure 3B**). Multivariable analysis was only performed for OS because the p-value for RFS did not meet the criteria (p-value <0.1) for all parameters. Multivariable analysis confirmed worse OS with *FLT3*-ITD (HR 1.51), ECOG 3–4 (HR 2.47), and age >60 years (HR 1.89), while better OS with age <30 (HR 0.45), age 30–60 (HR 0.56), and ELN favorable-risk classification (HR 0.66).

### Use of FLT3 inhibitor therapy and treatment in relapse/refractory AML

Two patients with newly diagnosed *FLT3*-ITD AML received midostaurin in combination with intensive chemotherapy as frontline therapy. Both were classified as intermediate-risk AML with normal cytogenetics and concurrent type A *NPM1* mutations. The first patient, a 27-year-old woman, was treated with a 7 + 3 induction regimen plus midostaurin, followed by four cycles of high-dose cytarabine (HiDAC) combined with midostaurin. She remained in complete remission on midostaurin maintenance after 11.1 months of follow-up. The second patient, a 52-year-old man, also received 7 + 3 plus midostaurin and achieved CR. He proceeded to consolidation with a single cycle of HiDAC and underwent allogeneic transplantation from a matched unrelated donor. Midostaurin maintenance was not continued post-transplant due to financial constraints. At 13.6 months of follow-up, he remained in remission. These two cases represent the only instances in this cohort where *FLT3*-targeted therapy was incorporated into first-line treatment.

Among 232 patients (64.4%) with relapsed or refractory AML, 77.2% of *FLT3*-ITD and 59.5% of wild-type *FLT3* patients had relapsed or failed initial therapy. *FLT3*-ITD mutations were redetected at relapse in all previously mutated patients and newly emerged in 58.2% of initially wild-type cases. Only 21.5% of relapsed/refractory patients received salvage chemotherapy. MEC was administered to 13 *FLT3*-ITD and 25 wild-type patients, yielding CR rates of 23.1% and 32.0%, respectively (p = 0.425). FLAG-Idarubicin achieved CR in 1/5 *FLT3*-ITD (20.0%) and 3/7 wild-type *FLT3* (42.9%) (p = 0.092). Notably, *FLT3* inhibitors were not administered in the relapsed/refractory setting.

## Discussion

The prevalence of *FLT3*-ITD mutation in our study (28.1%) is consistent with previous reports from Western countries, which range from 18-30% (1–4). Our findings confirm that *FLT3*-ITD AML is associated with distinct clinical and molecular characteristics such as higher white blood cell counts, bone marrow blast percentages, and co-occurrence with *NPM1* mutations. These features contribute to the aggressive nature of *FLT3*-ITD AML and underscore the need for more effective therapeutic strategies. Despite comparable use of induction chemotherapy and complete remission rates between *FLT3*-ITD and wild-type *FLT3* AML patients, the significantly shorter overall survival in the *FLT3*-ITD group highlights the dismal prognosis associated with this mutation. This is particularly concerning in the context of limited access to *FLT3* inhibitors and allogeneic stem cell transplantations in Thailand. Socioeconomic factors played a significant role in the decision to proceed with allogeneic stem cell transplantation in Thailand, particularly due to the limited number of transplant centers and challenges in healthcare reimbursement. Furthermore, a proportion of AML patients in our study received palliative care due to treatment limitations (18.8% of *FLT3*-ITD AML and 16.6% of wild-type *FLT3* AML), which was associated with poor survival outcomes.

Subgroup analysis further elucidated the prognostic impact of *FLT3*-ITD, with age, performance status, AML subtype, cytogenetics, and co-mutations influencing survival outcomes. The interaction between *FLT3*-ITD and other molecular abnormalities such as *NPM1* and *TP53* mutations highlights the complex biology of AML. A favorable prognosis was associated with the co-mutation of *NPM1* and *FLT3*-ITD. In contrast, the trend towards poorer outcomes in *FLT3*-ITD AML with *TP53* mutations emphasizes the need for targeted therapies that can overcome the resistance conferred by these co-occurring mutations.

*FLT3*-ITD mutation is a well-established adverse prognostic factor in AML, which confers a higher risk of relapse and shorter overall survival. Several studies have consistently demonstrated a negative impact of *FLT3*-ITD on the clinical outcomes of patients with AML. *FLT3*-ITD mutations are associated with a significantly lower complete remission rate, shorter relapse-free survival, and overall survival in patients with AML treated with intensive chemotherapy (4). Several studies have reported that *FLT3*-ITD mutations are independent predictors of inferior overall survival in AML patients, regardless of age or cytogenetic risk group, even in the context of a normal karyotype (5, 19). The presence of *FLT3*-ITD mutations, particularly those with a high allelic ratio, is a strong adverse prognostic factor for overall survival in AML patients receiving allogeneic stem cell transplantation (20, 21).

The findings of this study are largely consistent with previous reports on the clinical characteristics and outcomes of *FLT3*-ITD AML patients. The prevalence of *FLT3*-ITD mutation in our cohort (28.1%) is within the range reported in Western countries, which typically vary between 25-35% (1). Similar to other studies, we found that *FLT3*-ITD AML patients were more likely to have normal cytogenetics and *NPM1* mutations. Despite the comparable use of induction chemotherapy and complete remission rates between *FLT3*-ITD and wild-type *FLT3* AML patients, the significantly shorter overall survival in the *FLT3*-ITD group is consistent with the dismal prognosis associated with this mutation reported in previous studies (4, 5). The comparable relapse-free survival between *FLT3*-ITD and wild-type *FLT3* AML patients in our study differs from some previous reports that have shown a higher risk of relapse in *FLT3*-ITD AML (4, 5). However, our findings suggest that the survival disadvantage of *FLT3*-ITD AML may be driven by factors other than relapse, such as primary refractory disease or early mortality. Although allogeneic stem cell transplantation improved OS in *FLT3*-ITD AML patients, it did not significantly prolong RFS. This finding may be explained by the high relapse risk associated with *FLT3*-ITD disease, the absence of post-transplant FLT3 inhibitor maintenance, lack of measurable residual disease (MRD) monitoring, and the limited sample size which may have reduced the statistical power to detect the difference. This finding underscores the need for novel therapies to overcome the inherent resistance of *FLT3*-ITD AML to conventional chemotherapy. The interaction between *FLT3*-ITD and other molecular abnormalities such as *NPM1* and *TP53* mutations has been reported in several studies. The favorable prognosis associated with the co-mutation of *NPM1* and *FLT3*-ITD in our study is consistent with previous findings that *NPM1* mutation may mitigate the adverse impact of *FLT3*-ITD (5, 19). In contrast, the trend towards poorer outcomes in *FLT3*-ITD AML with *TP53* mutations in our study emphasizes the need for targeted therapies that can overcome the resistance conferred by these co-occurring mutations (5, 22). The subgroup analysis in our study further elucidated the prognostic impact of *FLT3*-ITD, with age, performance status, AML subtype, cytogenetics, and co-mutations influencing the survival outcomes.

This study has several strengths and limitations that provide valuable insights into the real-world clinical practice of managing FLT3-ITD AML in Thailand. A key strength is the nationwide scope of the study, which included data from multiple institutions across the country, thereby enhancing the generalizability of the findings. The real-life information gathered reflects actual clinical practices and treatment outcomes in Thailand, highlighting the importance of mutational testing in guiding treatment decisions. These data can inform national policies regarding the implementation of precision medicine approaches, particularly in the context of FLT3-ITD mutations. However, several limitations should be acknowledged. The limited use of FLT3 inhibitors in newly diagnosed and relapsed/refractory FLT3-ITD AML may contribute to the poor survival outcomes observed. Furthermore, only approximately 10% of patients underwent allogeneic stem cell transplantation, which may also explain the inferior survival rates in this cohort. The absence of allelic ratio measurement and measurable residual disease (MRD) monitoring limits the depth of prognostic assessment. Variability in mutation testing methods across institutions may also have affected the consistency of molecular data.

## Conclusions

In summary, this real-world nationwide registry confirms the poor prognosis of *FLT3*-ITD AML in settings with limited access to FLT3 inhibitors and allogeneic transplantation. The findings highlight the need for broader access to targeted therapies and standardization in diagnostic practices. Future studies should integrate MRD tracking and comprehensive genomic profiling to enhance treatment strategies and improve outcomes for AML patients in resource-constrained regions.

## Data Availability

The raw data supporting the conclusions of this article will be made available by the authors, without undue reservation.
